# LATPS, a novel prognostic signature based on tumor microenvironment of lung adenocarcinoma to better predict survival and immunotherapy response

**DOI:** 10.3389/fimmu.2022.1064874

**Published:** 2022-11-24

**Authors:** Jihong Huang, Lu Yuan, Wenqi Huang, Liwei Liao, Xiaodi Zhu, Xiaoqing Wang, Jiaxin Li, Wenyu Liang, Yuting Wu, Xiaocheng Liu, Dong Yu, Yunna Zheng, Jian Guan, Yongzhong Zhan, Laiyu Liu

**Affiliations:** ^1^ Chronic Airways Diseases Laboratory, Department of Respiratory and Critical Care Medicine, Nanfang Hospital, Southern Medical University, Guangzhou, China; ^2^ Department of Radiation Oncology, Nanfang Hospital, Southern Medical University, Guangzhou, China; ^3^ Department of Blood Transfusion, Ganzhou People’s Hospital, Ganzhou, China

**Keywords:** immunotherapy, prognosis, immune infiltration, tumor microenvironment, LUAD

## Abstract

**Background:**

Clinically, only a minority of patients benefit from immunotherapy and few efficient biomarkers have been identified to distinguish patients who would respond to immunotherapy. The tumor microenvironment (TME) is reported to contribute to immunotherapy response, but details remain unknown. We aimed to construct a prognostic model based on the TME of lung adenocarcinoma (LUAD) to predict the prognosis and immunotherapy efficacy.

**Methods:**

We integrated computational algorithms to describe the immune infiltrative landscape of LUAD patients. With the least absolute shrinkage and selection operator (LASSO) and Cox regression analyses, we developed a LUAD tumor microenvironment prognostic signature (LATPS). Subsequently, the immune characteristics and the benefit of immunotherapy in LATPS-defined subgroups were analyzed. RNA sequencing of tumor samples from 28 lung cancer patients treated with anti-PD-1 therapy was conducted to verify the predictive value of the LATPS.

**Results:**

We constructed the LATPS grounded on four genes, including UBE2T, KRT6A, IRX2, and CD3D. The LATPS-low subgroup had a better overall survival (OS) and tended to have a hot immune phenotype, which was characterized by an elevated abundance of immune cell infiltration and increased activity of immune-related pathways. Additionally, tumor immune dysfunction and exclusion (TIDE) score was markedly decreased in the LATPS-low subgroup, indicating an enhanced opportunity to benefit from immunotherapy. Survival analysis in 28 advanced lung cancer patients treated with an anti-PD-1 regimen at Nanfang hospital revealed that the LATPS-low subgroup had better immunotherapy benefit.

**Conclusion:**

LATPS is an effective predictor to distinguish survival, immune characteristics, and immunotherapy benefit in LUAD patients.

## Introduction

Immunotherapy has dramatically revolutionized the landscape of non-small cell lung cancer (NSCLC) treatment (1). Among the various immunotherapy, immune checkpoint inhibitors (ICIs) reactivate the immune system to eliminate cancer cells, exhibiting a durable anti-tumor response in NSCLC patients (2, 3). However, not all NSCLC patients respond to ICIs treatment. The overall response rate (ORR) was only about 40% in PD-L1 > 50% cases (4, 5). Multiple reported factors including PD-L1, TMB, and MSI can’t efficiently predict immunotherapy response (6). Thus, new biomarkers are urgently needed.

Recently, the tumor microenvironment (TME) was demonstrated to exhibit a strong influence on the response to ICIs treatment (7, 8). Jiang P et al. constructed a tumor immune dysfunction and exclusion (TIDE) model based on the status of T cell dysfunction and exclusion. The TIDE model had a higher accuracy for predicting the immunotherapy response of advanced NSCLC compared with traditional PD-L1 expression and TMB (9). However, the TIDE model needs to conduct whole transcriptome sequencing of the tumor samples. Besides, the TIDE model only focused on the T cells’ status, which may not be insufficient to reflect the complexity of the TME in patients with NSCLC.

NSCLC accounts for nearly 85% of lung cancer and lung adenocarcinoma (LUAD) is the most common pathological type, making up approximately 40% of lung cancers (1). Thus, a deeper understanding of the TME might help to discover novel biomarkers for immunotherapy in LUAD. In the present study, we sought to explore the immune landscape in LUAD using the CIBERSORT and ESTIMATE algorithms, screen out differently expressed genes and construct a LUAD tumor microenvironment prognostic signature (LATPS). Subsequently, we explored the clinical value of the LATPS in predicting survival and immunotherapeutic benefits in LUAD patients.

## Materials and methods

### Patients and data collection

The RNA sequencing data and corresponding clinical annotations were retrieved from The Cancer Genome Atlas (TCGA) database (https://portal.gdc.cancer.gov/). Microarray profiles were downloaded from Gene Expression Omnibus (GEO) (https://www.ncbi.nlm.nih.gov/geo/). We collected 1088 LUAD patients (GSE42127, GSE72094, and TCGA-LUAD) and combined them into a meta cohort after normalization (10) to generate the LATPS.

To evaluate the predictive value of the LATPS for immunotherapy benefits, three independent immunotherapy cohorts, including two NSCLC cohorts who received anti-PD-1 treatment (GSE135222, GSE126044), 28 advanced NSCLC patients with intervention of anti-PD-1 therapy at Nanfang Hospital (Guangzhou, China) from January 2019 to June 2021, were chosen to verify the predictive value of the constructed LATPS for immunotherapy benefits. The detailed clinical characteristics are presented in **Supplementary Table 1**. In Nanfang Hospital cohort, Patients were eligible for enrolment if they were aged ≥18 years, diagnosed with advanced NSCLC, had an Eastern Cooperative Oncology Group (ECOG) performance status score of 0 or 1. Exclusion criteria included: unstable or untreated central nervous system metastases, uncontrolled infection, ongoing corticosteroid therapy over 10 mg prednisone per day, active autoimmune disease within the past 2 years, discontinued to received ICIs due to serious ICIs-related adverse events (IRAs), and those who lost of follow-ups. The patients were treated with anti-PD-1 therapy every 3 weeks as a cycle. Tumor response was assessed every 2 cycles according to the Response Evaluation Criteria in Solid Tumors (RECIST), version 1.1 (11). Archived formalin-fixed, paraffin embedded (FFPE) tumor samples of the 28 NSCLC patients were collected prior to receiving immunotherapy. Before sample collection, it was approved by the Ethics Committee of Nanfang Hospital. To validate the survival classification and predictive capability of the LATPS, other four independent LUAD cohorts, including GSE29016 (n=38), GSE31210 (n=226), GSE41271 (n=182), and GSE50081 (n=127) were applied as external validation cohorts.

### RNA sequencing and data processing

The RNA was first extracted from FFPE samples and quantified on a Qubit 3.0/4.0, then it was assessed on a 2100 Bioanalyzer. Next, a part of total RNA (50 ng) was used with the SMARTer Stranded Total RNA-Seq Kit v2 according to the low-throughput protocol. We applied the Illumina NovaSeq 6000 Sequencing System to conduct RNA-seq libraries paired-end sequencing after PCR enrichment and purification. To ensure data quality, we used Trimmomatic (12), RSeQC (13), and bowtie2 (14) to preprocess the raw reads and obtain clean reads, which were used for subsequent analyses. Based on default parameters, we used FeatureCounts (15) to evaluate the expression level of each gene. All the sequencing data used in this study passed the quality control, with the data screening threshold set at greater than 3 G, and a uniquely mapping rate greater than 60%.

### Identification of differentially expressed genes and functional enrichment analysis

The abundance of infiltrated immune cells in LUAD samples was evaluated based on the LM22 gene signature with the “CIBERSORT” package (16). We used the “ESTIMATE” package to assess the immune and stromal contents of each LUAD sample, which further generated TME scores, including ImmuneScore, StromalScore, and ESTIMATEScore. The ESTIMATEScore was calculated as the sum of ImmuneScore and StromalScore. Higher ESTIMATEScore refers to lower tumor purity (17). According to the CIBERSORT results, we performed consensus clustering with the “ConsensusClusterPlus” package (18). We applied the “km” algorithm based on “euclidean” distance of ConsensusClusterPlus package. Subsequently, an empirical cumulative distribution function (CDF) diagram and a delta area diagram were generated to visualize the clustering results, in which k represented the number of subgroups. We chose k = 3 as the optimal value for the delta area showed a significant reduction and CDF plateaued when k > 3, which classified LUAD patients into three TME subgroups. A consensus matrix was generated to demonstrate the clustering stability of the hierarchical clustering results. Principal component analysis (PCA) was used to visualize the clustering pattern. DEGs among different TME subgroups were identified using the “Limma” package with the screening threshold set at a p-value< 0.05 and an absolute log2FoldChange > 1. “Boruta” package was applied to reduce superfluous genes. We conducted gene ontology (GO) enrichment analysis utilizing the “clusterProfiler” package (19). GO terms with p-value< 0.05 were considered statistically significant.

### Constructing the LATPS for patients with LUAD

We screened out 1035 LUAD patients (the total cohort) with matched survival information from the meta cohort. Then, the total cohort was randomly divided into a training cohort and a test cohort at a ratio of 1:1. We used the training cohort to identify prognostic genes and construct the LATPS. Firstly, we used univariate Cox regression analysis to screen out the significant prognostic genes from the DEGs (p-value< 0.01). Secondly, to minimize overfitting (20), we performed LASSO analysis using the “glmnet” package. Finally, after filtration using LASSO analysis, we established the LATPS based on four hub genes filtered by Multivariate Cox regression analysis. Subsequently, we calculated the LATPS score as follows:


$$\text{LATPS score }=\sum_{\text{i}}Coefficient\;of\;gene(i)\;\times \;Expression\;of\;gene\;(i)$$

*Coefficient of gene (i)* represents the regression coefficients of the four hub genes in the Cox model and *Expression of gene (i)* means the expression value of the four hub genes for patients with LUAD. Thereafter, we classified the patients into a LATPS-high subgroup and a LATPS-low subgroup according to the median LATPS scores. Moreover, we conducted survival analysis using “survival” and “survminer” packages. To evaluate the predictive power and capability of the LATPS, Time-dependent receiver operating characteristic (ROC) in the “timeROC” package was analyzed. Furthermore, we performed a prognostic meta-analysis to evaluate the comprehensive predictive significance of LATPS in four validation cohorts (n=573) using the “meta” R package.

### Analyzing the predictive value of the LATPS for immunotherapy response

We applied single sample gene set enrichment analysis (ssGSEA) algorithm to quantify the relative abundance of the immune cell infiltration in each LUAD sample using the gene set variation analysis (GSVA) package. Twenty eight immune cell subpopulations gene signatures were obtained from a previous study (21) and the other 24 types of tumor-infiltrating immune cells (TIICs) gene signatures were downloaded from the Immune Cells Abundance Identifier (ImmuCellAI) database. We then performed GSVA to estimate the variation of pathway activity over a sample population in an unsupervised manner based on the “GSVA” package (22). We obtained the twenty five immune-related pathways gene signatures from a previous study (23). The Spearman method was utilized to analyze the correlation between LATPS score and immune-related pathways or immune cell infiltration level. Results were filtered by setting a p-value< 0.05 as a threshold and were visualized using lollipop plots. Thereafter, we scored LUAD patients using the TIDE algorithm online (http://tide.dfci.harvard.edu/). Additionally, we performed survival and ROC analyses in three independent cohorts who received immunotherapy to investigate the potential value of the LATPS to predict immunotherapy benefits.

### Establishing a nomogram signature

We collected clinicopathological factors integrated with transcriptome profile of LUAD patients. Then we performed univariate and multivariate Cox regressions to determine whether the LATPS model was an independent prognostic factor. We employed the “rms” and “foreign” packages to establish a predictive nomogram on the basis of the clinicopathological factors and LATPS score. Subsequently, calibration curve and ROC curve analyses were used to assess the predictive precision of the nomogram.

### Statistical analysis

The Mann-Whitney U test was employed to compare continuous variables between two groups. Kruskal–Wallis tests were used to conduct difference comparisons of three or more groups (24). The Chi-squared test was carried out to compare categorical variables between two groups. Survival curve analysis was conducted using the Kaplan–Meier method and log-rank tests were used to identify significant differences among subgroups. A p-value< 0.05 was considered statistically significant. All analyses were processed with R version 4.0.2 and its appropriate packages.

## Results

### Characterization of immune cell landscape in LUAD

**Figure 1:**
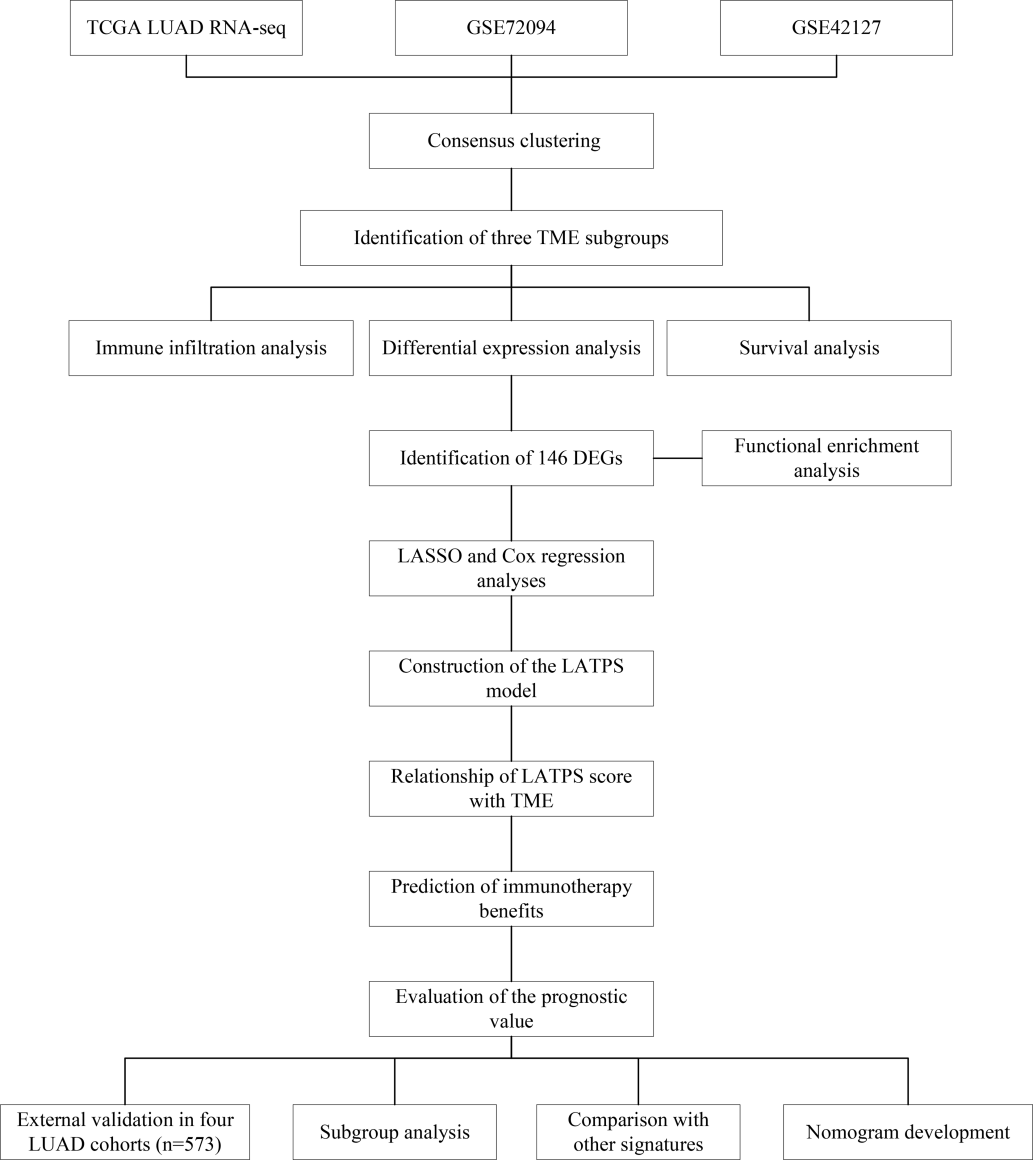
The workflow chart of this study.

**Figure 2:**
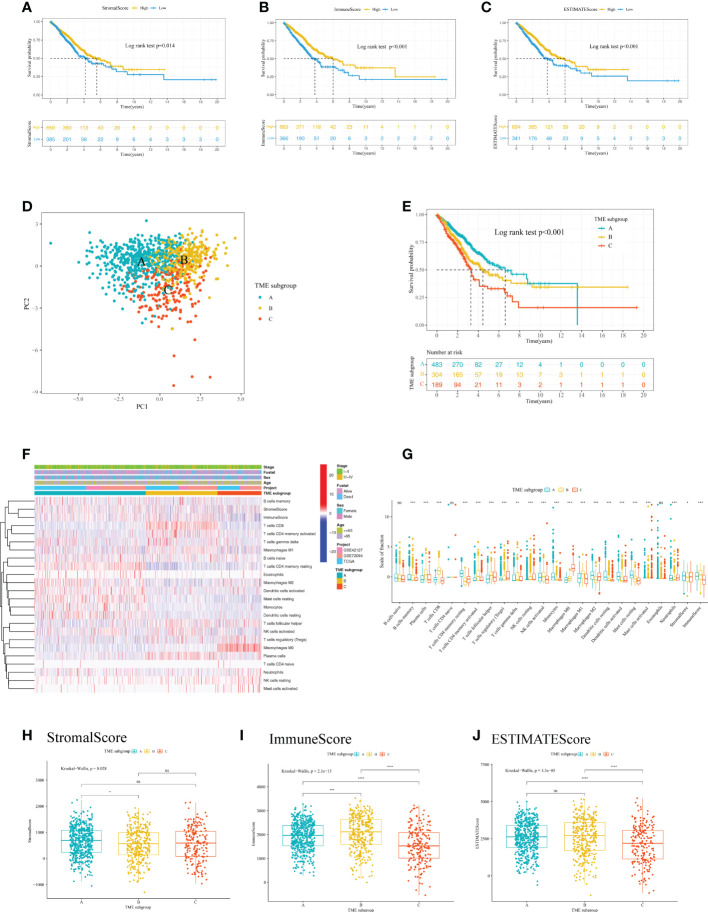
Analysis of the immune cell infiltration and TME scores of patients with LUAD. Kaplan–Meier curve analysis of the OS for different levels of **(A)** StromalScore, **(B)** ImmuneScore, and **(C)** ESTIMATEScore. **(D)** PCA for the immune cell infiltration level of the three TME subgroups, showing a remarkable difference in immune cell infiltration levels between different subgroups. **(E)** Kaplan–Meier curve analysis for the OS of patients with LUAD in different TME subgroups. **(F)** Heatmap of the 22 TIICs in different LUAD cohorts. Rows represent TIICs, and columns indicate LUAD samples. **(G)** The fraction of 22 TIICs, StromalScore, and ImmuneScore were compared between different TME subgroups using the Kruskal-Wallis test. The Kruskal–Wallis test was used to compare the statistical difference of **(H)** StromalScore, **(I)** ImmuneScore and **(J)** ESTIMATEScore of the three TME subgroups. *p< 0.05; **p< 0.01; ***p< 0.001; ****p< 0.0001; ns, no significance. LUAD, lung adenocarcinoma; TME, tumor microenvironment; OS, overall survival; PCA, principal component analysis; TIIC, tumor infiltrating immune cell.

The workflow chart of our study is shown in **Figure 1**. LUAD samples (n = 1088) from GSE72094, GSE42127, and TGCA-LUAD were combined into one meta-cohort after normalization. **Table 1** summarizes the baseline information of the patients with LUAD in different datasets. PCA was applied to visualize the overall expression pattern of the three LUAD cohorts before and after normalization (**Supplementary Figures 1A, B**). The ESTIMATE algorithm then generated TME scores, including StromalScore, ImmuneScore, and ESTIMATEScore. Survival analyses showed that TME score-high patients had better OS, indicating that the TME may influence the OS of LUAD patients (**Figures 2A-C**).

**Table 1:** Clinical characteristics of patients with LUAD in each dataset.

Characteristics		Dataset		
		GSE42127	GSE72094	TCGA
Platform (%)		GPL6884	GPL15048	IlluminaHiSeq
Patients (n)		133	442	513
Age (%)	≤65	65 (48.9)	127 (28.7)	238 (46.4)
	>65	68 (51.1)	294 (66.5)	256 (49.9)
	NA	0 (0.0)	21 (4.8)	19 (3.7)
Sex (%)	Female	65 (48.9)	240 (54.3)	276 (53.8)
	Male	68 (51.1)	202 (45.7)	237 (46.2)
Stage (%)	I	89 (66.9)	265 (60.0)	274 (53.4)
	II	22 (16.5)	69 (15.6)	121 (23.6)
	III	20 (15.0)	63 (14.3)	84 (16.4)
	IV	1 (0.8)	17 (3.8)	26 (5.1)
	NA	1 (0.8)	28 (6.3)	8 (1.6)
Survival (%)	Alive	90 (67.7)	298 (67.4)	326 (63.5)
	Dead	43 (32.3)	122 (27.6)	187 (36.5)
	NA	0 (0.0)	22 (5.0)	0 (0.0)

NA, not available.

To further analyze the immune cell landscape of LUAD patients, we first calculated the abundance of 22 immune cell subpopulations of each LUAD sample using the CIBERSORT algorithm. We then performed unsupervised clustering to categorize LUAD patients into three TME subgroups according to the CIBERSORT results. (**Supplementary Figures 2A, B**). The consensus matrix showed that when k = 3, there was little crossover between LUAD samples (**Supplementary Figure 2C**). In addition, PCA indicated a marked difference in immune cell infiltration levels among the TME subgroups (**Figure 2D**). To explore the clinical significance of the TME subgroups, we performed a survival analysis. As a result, the three TME subgroups showed a significant difference in OS (log-rank test, P<0.001) (**Figure 2E**).

We next aimed to investigate the distribution of tumor-infiltrating immune cells (TIICs) among TME subgroups. A heatmap was generated to visualize the distribution of TIICs (**Figure 2F**). TME subgroup A was marked by higher-level infiltration of monocytes, M2 macrophages, activated dendritic cells, resting dendritic cells, resting mast cells, memory B cells, and memory resting CD4^+^ T cells. TME subgroup B was characterized by higher-level infiltration of plasma cells, CD8^+^ T cells, memory activated CD4^+^ T cells, follicular helper T cells, gamma delta T cells, activated natural killer cells, and M1 macrophages. TME subgroup C was featured by a notable elevated regulatory T cell (Treg) and M0 macrophage infiltration. A boxplot further revealed the different distribution of TIICs in the three TME subgroups (**Figure 2G**). Additionally, we observed a higher StromalScore in TME subgroup A (P<0.05) (**Figure 2H**), a greater ImmuneScore in TME subgroup B (P<0.05) (**Figure 2I**), and a lower ESTIMATEScore in TME subgroup C (P<0.05) (**Figure 2J**), suggesting differences in tumor purity among the three TME subgroups.

### Construction of the LATPS

To obtain quantitative indexes of immune cell landscape in LUAD patients, differential expression analysis to identify the transcriptome variations among the TME subgroups was performed using the Limma package, which identified 149 DEGs. Volcano plots were constructed to show the results of pairwise comparison between the TME subgroups (**Supplementary Figures 2D-F**). We then performed the Boruta method to reduce redundant genes, leaving 146 candidate DEGs. By using the clusterProfiler package, GO enrichment analysis of the DEGs was carried out, and it was found that they were significantly enriched in humoral immune response, T cell activation, and extracellular organization (**Supplementary Figures 2G**).

**Figure 3:**
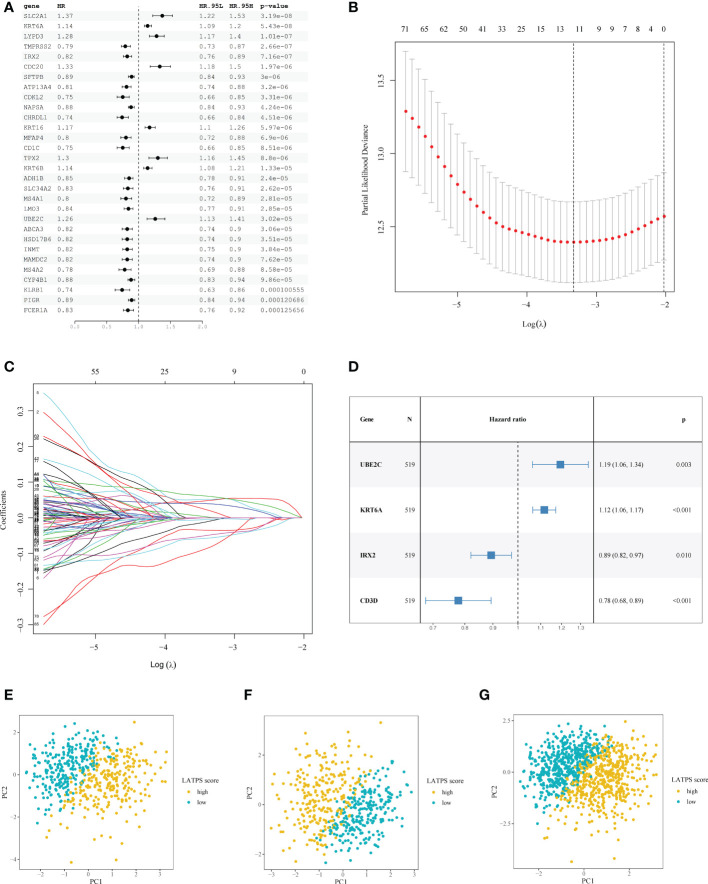
Construction of the LATPS. **(A)** Forest plot presenting the top 30 significant genes from the univariate Cox analysis results. **(B)** A coefficient profile plot was generated against the log (lambda) sequence. Selection of the optimal parameter (lambda) in the LASSO model. **(C)** LASSO coefficient profiles of the 93 candidate prognostic genes. **(D)** Forest plot illustrating the multivariate Cox model results. PCA showing the distribution differences between the LATPS-high and LATPS-low subgroups of the **(E)** training, **(F)** test, and **(G)** total cohorts. LATPS, LUAD tumor microenvironment prognostic signature; LASSO, least absolute shrinkage and selection operator; PCA, principal component analysis.

Next, LUAD patients with complete prognostic information (the total cohort) were randomly divided into a training cohort (n = 519) and a test cohort (n = 516). There was no statistical difference in clinicopathological parameters between the training and test cohorts (**Table 2**). Univariate Cox regression analysis was conducted in the training cohort to further explore the prognostic value of the 146 candidate DEGs, which identified 93 genes that were associated significantly with survival (**Supplementary Table 2** **)**. The top 30 significant genes were shown in **Figure 3A**.

**Table 2:** Clinical characteristics of patients with LUAD in different dataset.

Characteristics		Dataset		p value
		Training cohort	Test cohort	
n		519	516	
Age (%)	<=65	207 (39.9)	214 (41.5)	0.736
	>65	306 (59.0)	298 (57.8)	
	NA	6 (1.2)	4 (0.8)	
Sex (%)	Female	270 (52.0)	287 (55.6)	0.272
	Male	249 (48.0)	229 (44.4)	
Stage (%)	I	318 (61.3)	295 (57.2)	0.102
	II	94 (18.1)	114 (22.1)	
	III	72 (13.9)	86 (16.7)	
	IV	27 (5.2)	15 (2.9)	
	NA	8 (1.5)	6 (1.2)	
Survival (%)	Alive	355 (68.4)	341 (66.1)	0.467
	Dead	164 (31.6)	175 (33.9)	

NA, not available.

To avoid overfitting of the candidate genes, LASSO analysis was performed and 12 genes were retained (**Figure 3B, C**). Multivariate Cox regression analysis was used to establish the prognostic signature and four hub genes, including *UBE2C* (encoding ubiquitin conjugating enzyme E2 C), *KRT6A* (encoding keratin 6A), *IRX2* (encoding iroquois homeobox 2), and *CD3D* (encoding CD3d molecule) were identified (**Figure 3D**). We scored each patient with LUAD with following formula: LATPS score = UBE2C*0.177738 + KRT6A*0.110354 + IRX2*(-0.112574) + CD3D*(-0.250127).

Moreover, PCA revealed markedly different distribution patterns of the four hub genes between the LATPS-high and LATPS-low subgroups in the training (**Figure 3E** and **Supplementary Figure 3A**), test (**Figure 3F** and **Supplementary Figure 3B**), and total cohorts (**Figure 3G** and **Supplementary Figure 3C**).

### Correlation between the LATPS and the TME

**Figure 4:**
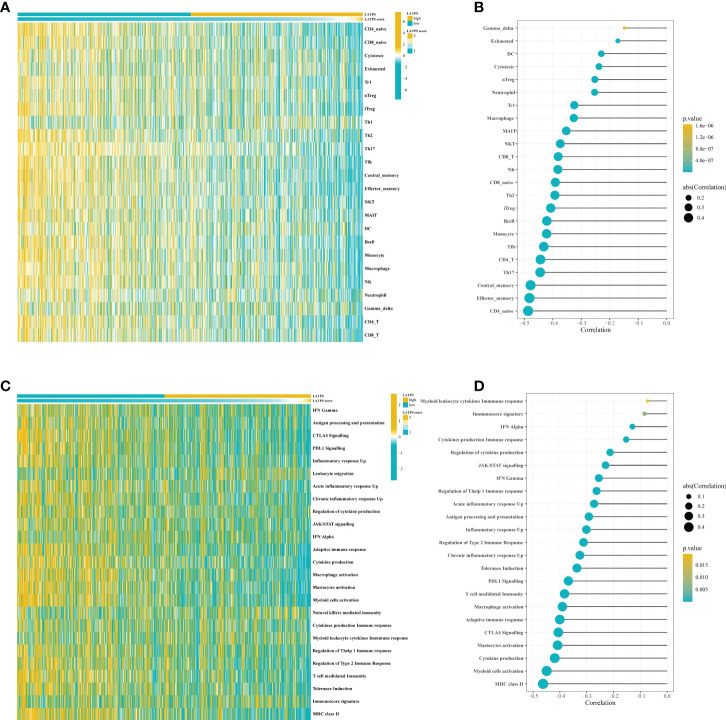
The LATPS score is associated with immune cell infiltration and immune activation. **(A)** Heatmap showing the LATPS score and relative abundance of 24 TIICs. **(B)** Lollipop plot showing the correlation between the LATPS score and the ssGSEA scores of 24 TIICs. **(C)** Heatmap presenting the LATPS score and GSVA scores of 25 immune-related pathway gene sets. **(D)** Lollipop plot presenting the correlation between the LATPS score and GSVA scores of 25 immune-related pathway gene sets. LATPS, LUAD tumor microenvironment prognostic signature; TIIC, tumor infiltrating immune cell; ssGSEA, single sample gene set enrichment analysis; GSVA, gene set variation analysis.

We then sought to explore the immune characteristics of the LATPS-defined subgroups. The ESTIMATE algorithm was used to estimate tumor purity in LUAD samples. Boxplots showed distinct distributions of StromalScore, ImmuneScore, and ESTIMATEScore between the LATPS subgroups (**Supplementary Figure 3D-F**). Notably, the ImmuneScore was significantly higher in the LATPS-low subgroup (Mann-Whitney U test, P<2.2e−16) (**Supplementary Figure 3E**). Immune activation and immune infiltration are pivotal components of the immune system; therefore, we evaluated the abundance of immune cells and the activation of immune-related pathways using the GSVA package. The heatmap showed that the LATPS-low patients had a higher infiltration level for most TIICs (**Figure 4A**). For further validation, a lollipop plot was constructed, which revealed that the LATPS score correlated negatively with the infiltration of most immune cells (**Figure 4B**).

Additionally, a heatmap showed that the majority of immune-related pathways were significantly enriched in the LATPS-low subgroup, comprising antigen processing and presentation, CTLA4 Signalling, and PDL1 Signalling (**Figure 4C**). The LATPS score was correlated negatively with the majority of immune-related pathways (**Figure 4D**). Collectively, these results suggested that the LATPS-low subgroup tended to be a hot immune phenotype and might benefit more from immunotherapy (23).

### The role of the LATPS in predicting immunotherapeutic benefits

**Figure 5:**
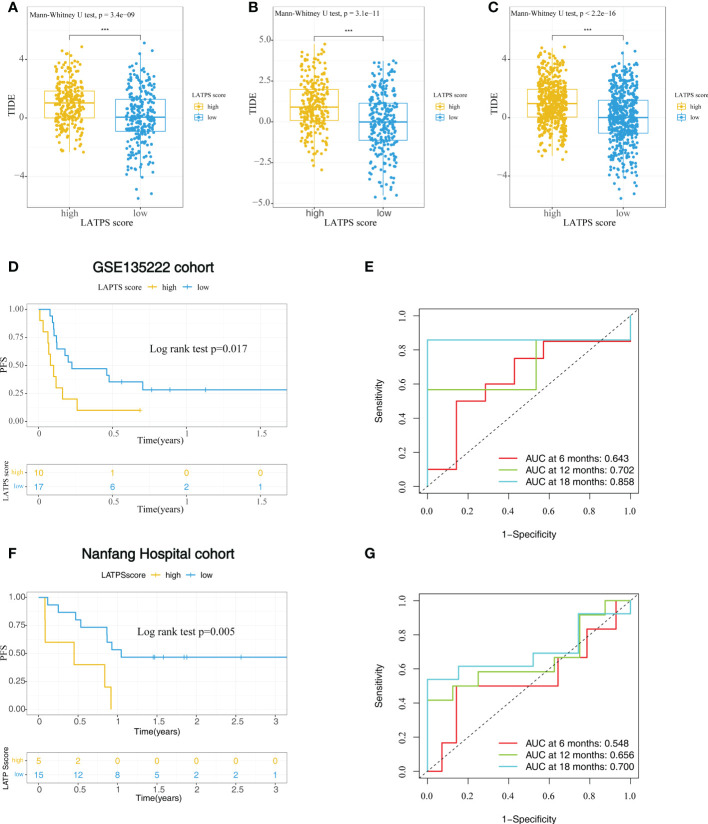
The role of the LATPS in the prediction of immunotherapeutic benefits. The relative distribution of TIDE was compared between the LATPS-high and LATPS-low subgroups in the **(A)** training, **(B)** test, and **(C)** total cohorts. **(D, E)** Kaplan–Meier curve and ROC curve analyses of the LATPS for predicting immunotherapy benefits in GSE135222 cohort. **(F, G)** Kaplan–Meier curve and ROC curve analyses of the LATPS for predicting immunotherapy benefits in Nanfang Hospital cohort. LATPS, LUAD tumor microenvironment prognostic signature; ROC, receiver operating characteristic.

To further explore whether the LATPS could distinguish potential immunotherapeutic benefits for different subgroups, we scored each LUAD sample using TIDE algorithm and visualized the distribution of the results as waterfall plots (**Supplementary Figure 3G-I**). A higher TIDE score represents a greater possibility of immune dysfunction and immune evasion, indicating that the patients would receive less benefit from immunotherapy (9). Notably, the LATPS-low patients had a lower TIDE score, suggesting that these patients might achieve a better immunotherapy response (**Figure 5A-C**).

To verify the above speculation, we assessed the predictive value in NSCLC cohorts receiving anti-PD-1 treatment, including GSE135222, GSE126044 and Nanfang Hospital cohorts. As a result, we could find that LATPS-low patients had better progression-free survival (PFS) in GSE135222 cohort (log-rank test, P=0.017) (**Figure 5D**) and Nanfang Hospital cohort (log-rank test, P=0.005) (**Figure 5F**). The AUC of LATPS for predicting immunotherapy benefits was 0.643 at 6 months, 0.702 at 12 months, and 0.858 at 18 months follow-up in GSE135222 cohort (**Figure 5E**). As for Nanfang Hospital cohort, the AUC was 0.548 at 6 months, 0.656 at 12 months, and 0.700 at 18 months follow-up, respectively (**Figure 5G**). Moreover, the LATPS score had the potential to distinguish patients with different anti-PD-1 responses (Mann-Whitney U test, P=0.052) (**Supplementary Figure 4A**). ROC analysis revealed that the LATPS had a promising accuracy to predict immunotherapy response in the GSE126044 cohort, with an AUC of 0.818. (**Supplementary Figure 4B**). These findings strongly suggested that the LATPS is a promising prognostic biomarker that can predict immunotherapy benefits.

### Exploring and validating the prognostic value of the LATPS

**Figure 6:**
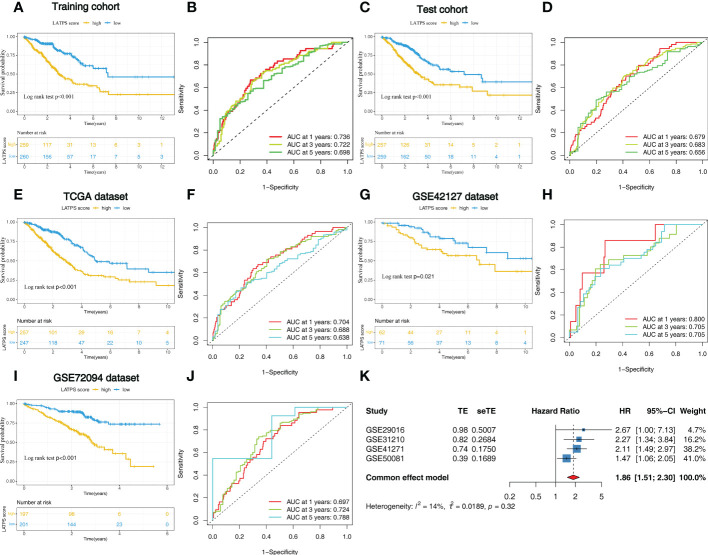
Identification of the LATPS in the training, test, and external validation cohorts. **(A, B)** Kaplan–Meier curve and the ROC curve for training cohort. **(C, D)** Kaplan–Meier curve and the ROC curve for test cohort. **(E, F)** Kaplan–Meier curve and the ROC curve for TCGA dataset. **(G, H)** Kaplan–Meier curve and the ROC curve for GSE42127 dataset. **(I, J)** Kaplan–Meier curve and the ROC curve for GSE72094 dataset. **(K)** Results of the prognostic meta-analysis on the basis of four external LUAD cohorts. LATPS, LUAD tumor microenvironment prognostic signature; ROC, receiver operating characteristic.

To further explore the prognostic value of the LATPS in patients with LUAD, we performed survival analysis in the training cohort. As it revealed that patients in the LATPS-low subgroup had a significantly better OS (log-rank test, P<0.001) (**Figure 6A**). We then performed a Time-dependent ROC analysis to evaluate the accuracy of the LATPS. The areas under the curves (AUCs) of this signature for 1-, 3-, and 5- year OS were 0.736, 0.722, and 0.698, respectively (**Figure 6B**).

We then aimed to interrogate whether the prognostic predictive power of the LATPS is of robustness, the patients were divided into LATPS-high and LATPS-low subgroups in the test cohort according to the median LATPS score used in the training cohort. Consistent with the results in the training cohort, survival analysis showed that the LATPS-low subgroup experienced a better outcome than the LATPS-high subgroup in the test cohort (log-rank test, P< 0.001) (**Figure 6C**) and the AUC at 1, 3, and 5 years was 0.679, 0.683, and 0.656 in the test cohort (**Figure 6D**). Meanwhile, we assessed the predictive value of LATPS in internal independent datasets, including the TCGA dataset, GSE42127 dataset, and GSE72094 dataset. The results from the above datasets showed the same trend in OS, with great significance (log-rank test, P< 0.001, P = 0.021, P< 0.001), and the AUC at 1, 3, and 5 years was 0.704, 0.688, and 0.638 in TCGA dataset; 0.800, 0.705, 0.705 in GSE42127 dataset; 0.697, 0.724, and 0.788 in GSE72094 dataset, respectively (**Figure 6E-J**). Moreover, we performed a prognostic meta-analysis to assess the integrated predictive significance of LATPS. The selected fixed effects model of the meta-analysis showed that the LATPS is a significant predictor of OS in external LUAD patients (HR: 1.86, 95%CI: 1.51-2.30, P< 0.001) (**Figure 6K**).

### The association between the LATPS and clinical characteristics

**Figure 7:**
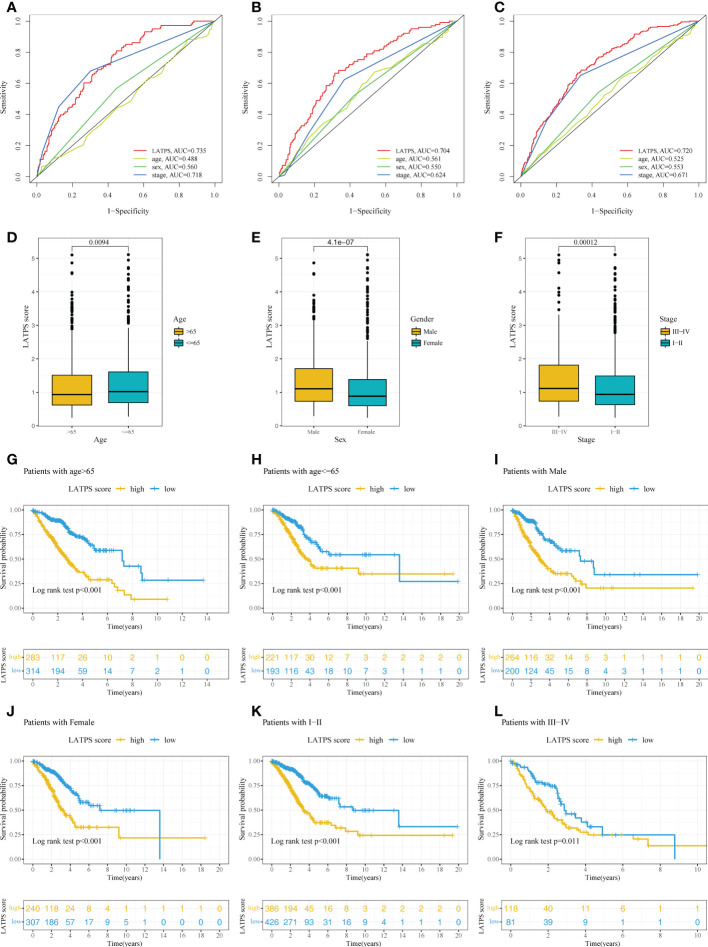
Confirmation of the LATPS *via* stratification of patients based on specific demographic and clinical features. Time-dependent ROC curve analysis of the LATPS score and clinicopathological factors to assess the predictive capacity of the LATPS in the **(A)** training, **(B)** test, and **(C)** total cohorts. **(D-F)** Boxplot showing the relationships between the LATPS score and clinicopathologic factors for all patients with LUAD. **(G-L)** Kaplan–Meier curve analysis for patients of **(G)** age > 65, **(H)** age ≤ 65, **(I)** Male, **(J)** Female, **(K)** Stage I–II, **(L)** Stage III–IV in the LATPS-high and LATPS-low subgroups. LATPS, LUAD tumor microenvironment prognostic signature; LUAD, lung adenocarcinoma; ROC, receiver operating characteristic.

Next, univariate and multivariate Cox regression analyses were conducted to assess whether the LATPS score could predict patients’ prognoses independently. The results indicated that both the stage and LATPS score can independently predict patients’ prognoses (**Table 3**). Time-dependent ROC curves analysis to further compare the predictive capacity between the LATPS score and clinicopathological factors revealed that the LATPS score had a higher AUC than the other factors (**Figures 7A-C**). This implied that the LATPS can more precisely predict the patient’s prognosis than the other clinicopathological factors. Boxplots were generated to describe the distribution of the LATPS score *via* stratification of patients based on age, sex, and stage. Results showed that the LATPS score was notably elevated in males, patients aged below 65 years, and in stage III–IV (**Figures 7D-F**). Moreover, stratified survival analysis revealed that LATPS-low patients were linked to better OS (**Figures 7G-L**), which agreed with our result in the training cohort.

**Table 3:** Univariate and multivariate Cox regression analysis in training, test, and total cohorts.

Variables	Univariate analysis				Multivariate analysis			
	HR	HR.95L	HR.95H	P value	HR	HR.95L	HR.95H	P value
Training cohort								
Age	1.004	0.989	1.020	0.574	1.002	0.986	1.018	0.841
Sex	1.256	0.921	1.715	0.150	1.081	0.781	1.497	0.637
Stage	1.863	1.615	2.149	0.000	1.745	1.506	2.022	0.000
LATPS score	1.866	1.617	2.153	0.000	1.820	1.559	2.125	0.000
Test cohort								
Age	1.018	1.002	1.034	0.031	1.019	1.004	1.035	0.015
Sex	1.362	1.008	1.839	0.044	1.144	0.837	1.566	0.399
Stage	1.448	1.241	1.690	0.000	1.462	1.248	1.711	0.000
LATPS score	1.570	1.350	1.826	0.000	1.550	1.319	1.822	0.000
Total cohort								
Age	1.011	1.000	1.022	0.056	1.012	1.001	1.023	0.039
Sex	1.296	1.045	1.609	0.018	1.070	0.859	1.332	0.547
Stage	1.655	1.491	1.838	0.000	1.608	1.445	1.788	0.000
LATPS score	1.711	1.542	1.897	0.000	1.700	1.524	1.897	0.000

### Comparison with other published LUAD signatures and construction of a nomogram signature

**Figure 8:**
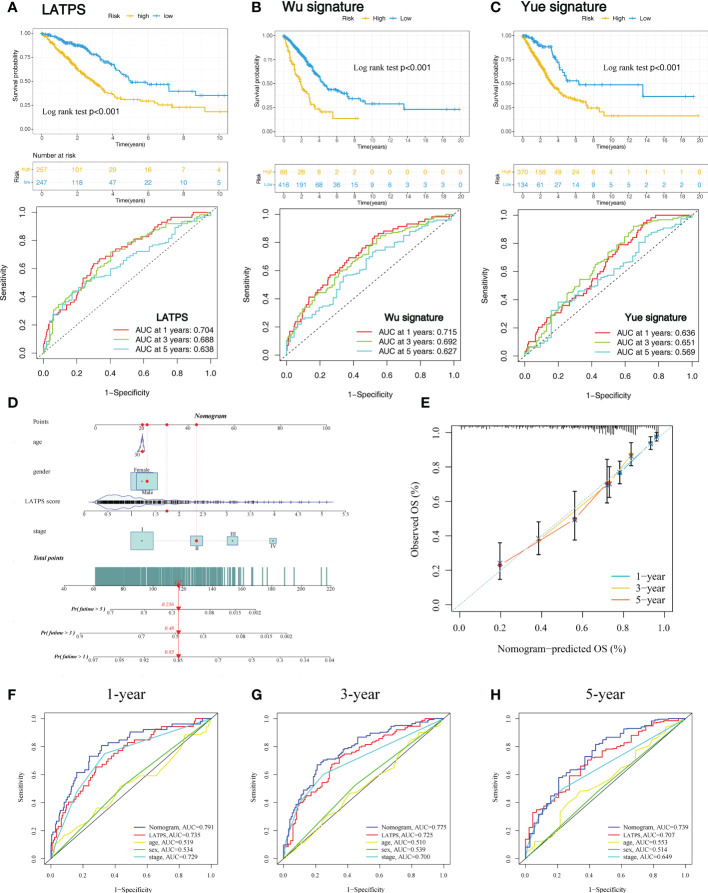
Comparison of the LATPS with other published gene signatures and construction of a nomogram. Kaplan–Meier curve and the ROC curve of **(A)** LATPS, **(B)** Wu signature, and **(C)** Yue signature. **(D)** Nomogram based on the LATPS and clinical information of patients with LUAD. **(E)** Calibration curve of the nomogram used for predicting OS at 1, 3, and 5 years. Time-dependent ROC curves analysis of the nomogram and clinicopathological factors in predicting **(F)** 1-, **(G)** 3-, and **(H)** 5-year OS. LATPS, LUAD tumor microenvironment prognostic signature; LUAD, lung adenocarcinoma; OS, overall survival; ROC, receiver operating characteristic.

To further evaluate the survival classification and predictive capacity of LATPS. We not only compared the LATPS with clinicopathological factors but also compared the predictive performance of two TME-based LUAD signatures. Wu signature was an 8-gene signature (25). Yue signature was a signature consisting of 3 genes (26). We applied Kaplan–Meier curve and the ROC curve analyses to assess the predictive efficacy of the above signatures. As a result, (LATPS, Wu signature, and Yue signature) had the same significant trend in survival, for patients in the low-risk group had better OS (log-rank test, P<0.001,p<0.001, p<0.001), and the AUC was 0.704, 0.715, 0.636 at 1 year; 0.688, 0.692, 0.651 at 3 years; and 0.638, 0.627, 0.569 at 5 years, respectively (**Figures 8A-C**).

Next, to assess the clinical utility of LATPS, a nomogram signature was established according to the clinicopathological factors and LATPS score in the training cohort. Each patient was scored according to their clinical features and LATPS score to predict survival probability (**Figure 8D**). Calibration curve analysis revealed that actual and nomogram-predicted OS corresponded well (**Figure 8E**). ROC curve analysis showed that the nomogram signature had more favorable predictive accuracy than other clinicopathological signatures (**Figures 8F-H**). Moreover, Calibration curve and ROC curve analyses of the nomogram signature in internal cohorts indicated that the nomogram signature was of favorable predictive capacity for OS (**Supplementary Figures 5A-H**). Collectively, these results suggested that the LATPS had clinical utility as a prognostic tool.

## Discussion

ICIs treatment only benefits a fraction of NSCLC patients with PD-L1 > 1% (5). Nevertheless, the IMpower132 study showed an OS benefit in PD-L1-negative patients treated with ICI therapy (27). Moreover, a previous study revealed that the accuracy of TMB in predicting the immunotherapy response for NSCLC is only about 60% (9). Therefore, conventional PD-L1 expression and TMB may not be enough to distinguish patients who would benefit from ICIs. Jiang P and Daniela ST pointed out that the status of T cells and the infiltration of T cells may be promising biomarkers for NSCLC treated with immunotherapy (9, 28). However, the TME of NSCLC is complicated and heterogeneous, which consists of various immune cells apart from T cells. Furthermore, taking into consideration that LUAD and lung squamous carcinoma (LUSC) were different in the tumor immune landscape (29), a deeper mining of the TME of LUAD may provide new insights for predicting immunotherapy response.

We analyzed the immune landscape in LUAD samples and identified three distinct TME subgroups. Notably, TME subgroup A was associated with the best OS and exhibited a significant increase in the infiltration of memory B cells, memory resting CD4^+^ T cells, monocytes, M2 macrophages, dendritic cells, and resting mast cells. Besides, TME subgroup B was associated with better prognosis, featured by an elevated infiltration of plasma cells, CD8^+^ T cells, gamma delta T cells, activated NK cells, M1 macrophages, and a higher ImmuneScore compared with TME subgroup C. Conversely, TME subgroup C was associated with the worst OS and was marked by a greater density of Tregs and M0 macrophages infiltration. Previous studies have shown a high Treg density was associated with poor prognosis in a variety of cancers, including lung cancer (30, 31). Higher infiltration of CD8^+^ T cells and M1 macrophages was related to better survival outcomes, which agrees with previous studies (32, 33). Thus, the immune cell infiltration pattern played an important role in patient’s prognosis, which would provide guidance to predict clinical outcomes.

Clinically, it is difficult to obtain the immune infiltration pattern of each LUAD patient. It needs to perform whole transcriptome sequencing (detect approximately 20,000 genes) of LUAD tumor samples to identify the TME subgroups, which would be expensive and impractical in clinical practice. Thus, we aim to construct a simple and efficient signature to reflect the immune infiltration pattern and predict the survival of LUAD patients based on the identified TME subgroups. Besides, we wanted to unravel the underlying biological characteristics of the three TME subgroups and screen out the key genes that may influence the OS of the distinct TME subgroups. Therefore, we explored the transcriptome variation among the TME subgroups. Subsequently, we identified 146 TME-related DEGs after performing differential expression analysis. GO functional enrichment analysis revealed that the DEGs were mainly associated with immune-related GO terms, including humoral immune response, regulation of cell killing and T cell activation. Studies have demonstrated the abundance and dysfunction of immune cells might affect antitumor immunity and immunotherapy response (9, 34, 35). Thus, our results indicated that imbalances in these immune-related functions or pathways might result in diverse clinical outcomes in patients with LUAD. Based on the expression of the 146 DEGs may help to distinguish different infiltration patterns and provide personalized treatment.

However, in the clinic, it would be impractical to determine the mRNA expression of the 146 TME-related DEGs. Therefore, we utilized computational algorithms to select hub genes and established a LUAD TME prognostic signature (LATPS), comprising four hub prognostic genes (*UBE2C*, *KRT6A*, *IRX2*, and *CD3D*). Reportedly, these four genes correlated with patient survival. Overexpression of *UBE2C* was reported as an independent risk factor associated with dismal outcomes in patients with lung cancer (36, 37). Reportedly, *KRT6A* is associated with cell proliferation and invasion, which drives cancer progression by upregulating glucose-6-phosphate dehydrogenase (G6PD) through MYC signaling pathway (38). Consistent with previous studies, our results revealed that both *UBE2C* and *KRT6A* were LUAD risk factors. Elevated expression of *IRX2* was linked with shorter OS in nasopharyngeal carcinoma (NPC) (39). Interestingly, we identified *IRX2* as a protective factor in LUAD; however, limited studies have focused on the role of *IRX2* in LUAD. For *CD3D*, its higher expression is related to a better outcome in colon cancer (40). Previous studies discovered that *CD3D* correlates highly with lymphocyte infiltration and is regarded as a promising therapeutic target (41, 42). In addition, PCA revealed that the mRNA expression pattern of the four hub genes could categorize patients with LUAD into two different subgroups, implying that there may be a difference in immune infiltration pattern and survival between the LATPS-defined subgroups.

ICIs have revolutionized the treatment of NSCLC and improved outcomes (43, 44). Therefore, understanding the response to immunotherapy may help to predict patients’ prognoses. Studies revealed that TIICs of the TME play a crucial role in immunotherapy response (7, 8). Besides, patients with an inflammatory phenotype or an immunity-high phenotype have a better prognosis and are thought to be more likely to benefit from immunotherapy (23, 45). Therefore, we further explored the immune infiltration landscape in the LATPS-defined subgroups. Interestingly, similar to previous studies, patients in the LATPS-low subgroup tended to be a hot immune phenotype, characterized by elevated immune cell infiltration and hyperactivated immune-related pathways. Thus, our results suggested that the LATPS is of potential predictive value in assessing immunotherapy response. Cancer immunotherapy using ICIs functions by blocking inhibitory signaling and reactivating cytotoxic T lymphocytes to attack cancer cells (46). Multiple factors affect immunotherapy effectiveness and few biomarkers have been developed to accurately assess the benefit of immunotherapy. Jiang P et al. identified the TIDE score, which quantifies two different mechanisms of tumor immune escape, including T cell dysfunction and exclusion. A patient with a lower TIDE score is likely to benefit from immunotherapy. The accuracy of the TIDE score for predicting immunotherapy response in NSCLC was about 80% (9). While the TIDE score was based on small samples of 21 NSCLC patients treated with immunotherapy and it was complicated to calculate, limiting its clinical application. We observed a lower TIDE score in the LATPS-low subgroup, which indicated that the LATPS might be useful for patient selection before ICI treatment.

To verify the predictive value of the LATPS in elevating ICI treatment benefits, we performed survival analysis in immunotherapy cohorts. In the GSE135222 cohort, 27 advanced NSCLC patients received anti-PD-1 therapy. As shown in **Figure 5D**, patients with lower LATPS score obtained longer PFS (log rank test, p = 0.017). In addition, we collected FFPE tumor samples of NSCLC patients treated with anti-PD-1 based therapy at Nanfang Hospital for RNA sequencing analysis. Among them, 20 patients with available survival information. Consistently, the LATPS-low subgroup got longer PFS than the LATPS-high subgroup (log rank test, p = 0.005), suggesting that the LATPS could distinguish different outcomes in patients who received immunotherapy. The AUC of LATPS for predicting immunotherapy benefits was higher in the GSE135222 cohort compared with the Nanfang Hospital cohort. Considering the sample size of Nanfang Hospital is smaller than the GSE135222 cohort, which may explain the lower ACU in the Nanfang Hospital cohort. Thus, further large scale immunotherapy cohorts are needed to verify our results. Moreover, ROC curves of the above two cohorts revealed that the LATPS is a potential predictor to predict immunotherapy benefits with an AUC of 0.548 to 0.858. Besides, it was evident that LATPS has better predictive accuracy at longer follow-ups according to the ROC curve analysis.

Subsequently, we further evaluated the clinical value of the LATPS for predicting immunotherapy response. In the GSE126044 NSCLC immunotherapy cohort, patients who responded to anti-PD-1 therapy had lower LATPS scores compared with none responders (Mann-Whitney U test, p = 0.052). Although it was not statistically significant, there was a trend that lower LATPS scores were more likely to benefit from immunotherapy. Besides, the GSE126044 was grounded on small numbers of samples, consisting of only 16 patients. Further large immunotherapy cohorts are needed to verify this hypothesis. The TIDE model has been reported to predict the outcome of NSCLC treated with first-line anti-PD1 or anti-CTLA4 antibodies with an AUC of about 0.80 (9). In the GSE126044 cohort, the AUC of LATPS for predicting immunotherapy response was 0.818, which was comparable with the TIDE model. Therefore, our results showed that the LATPS model could serve as a promising biomarker, which would facilitate the development of new avenues for personalized immune-intervention strategies. In addition, The TIDE model mainly focuses on the T cell status, which might be insufficient to reflect the complexity of the TME in LUAD. Besides, whole transcriptome sequencing of tumor samples is needed to generate the TIDE score, which is inconvenient to conduct in the clinic. Our LATPS model comprises only four genes, making it easier than the TIDE model to apply in clinical practice.

Next, we aimed to assess the survival classification and predictive efficacy of LATPS. Survival analysis revealed that LATPS-low patients had better prognoses than the LATPS-high subgroup in the training cohort, indicating that the LATPS was closely linked to LUAD survival. Furthermore, validation of the predictive accuracy of the LATPS using internal cohorts and stratification survival analysis demonstrated that the LATPS can more precisely predict the prognosis of LUAD compared with other clinicopathological factors. Moreover, univariate and multivariate Cox regression analyses identified the LATPS as an independent risk factor to predict patient prognosis, which was confirmed by the prognostic meta-analysis. Collectively, our results showed that the LATPS is a robust and generalizable predictor for survival in LUAD.

We also compared the LATPS with other previously published signatures (Wu signature (25) and Yue signature (26)), which were based on the TME of LUAD patients. ROC analysis demonstrated that the LATPS has a better predictive ability than Yue signature. Meanwhile, LATPS has a lower AUC for predicting OS at 1 and 3 years, but a higher AUC at 5 years compared with Wu signature. However, LATPS is a 4-gene signature, which is easier to conduct than the 8-gene signature (Wu signature) in the clinic. These results indicate that the overall performance of our LATPS is superior to others.

Several studies have constructed prognostic models to predict patients’ OS; however, few of them have been applied clinically (33, 47, 48). Nomograms can conveniently and efficiently estimate cancer prognosis, and are used widely in clinical cancer research (49). Thus, we established a nomogram according to the LATPS score and clinicopathological factors, which can be conveniently obtained in the clinic. Calibration curve analysis showed favorable accordance between nomogram-predicted and actual OS in the training cohort. Additionally, ROC curve analysis showed that the nomogram signature had an AUC of 0.791, which was higher than other clinicopathological models. Thus, our results suggested that the LATPS is a promising prognostic tool with clinical utility.

Conclusively, we applied integrated analysis to explore the TME of LUAD and constructed a LATPS, which can serve as a reliable tool to predict the prognosis and immunotherapy benefits of LUAD patients; however, further large scale studies are needed to validate the signature in LUAD cohorts treated with immunotherapy.

## Data availability statement

The data presented in the study are deposited in the Figshare database, which is available by visiting https://figshare.com/articles/dataset/Nanfang_hospital_NSCLC_immunotherapy_cohort/21564015. The processed data and R codes used during the current study are available from the corresponding author upon reasonable request.

## Ethics statement

The studies involving human participants were reviewed and approved by The Ethics Committee of Nanfang Hospital. The patients/participants provided their written informed consent to participate in this study. Written informed consent was obtained from the individual(s) for the publication of any potentially identifiable images or data included in this article.

## Author contributions

Conceptualization and design: LYL, JG, and YZZ. Data collection: XZ, YW, XL, DY, and YNZ. Methodology: XW and LWL. Data analysis: JH and XZ. Software: JH and LY. Generating of the Figure: JL and WL. Writing of the manuscript: JH and WH. Revision of the manuscript: JH, LY, and XZ. All authors contributed to the article and approved the submitted version.

## Funding

This work was supported by the National Natural Science Foundation of China [grant number 81870026], the President Foundation of Nanfang Hospital, Southern Medical University [grant number 2020C044], and the Clinical Research Program of Nanfang Hospital, Southern Medical University [grant numbers 2018CR019, 2018CR021, 2020CR025].

## Acknowledgments

We appreciated the TCGA, GEO, and ImmuCellAI databases for the availability of the original data used in this study.

## Conflict of interest

The authors declare that the research was conducted in the absence of any commercial or financial relationships that could be construed as a potential conflict of interest.

## Publisher’s note

All claims expressed in this article are solely those of the authors and do not necessarily represent those of their affiliated organizations, or those of the publisher, the editors and the reviewers. Any product that may be evaluated in this article, or claim that may be made by its manufacturer, is not guaranteed or endorsed by the publisher.

## Supplementary material

Supplementary Figure 1
**(A)** Principal component analysis showing the distribution differences of different LUAD cohorts before removing batch effects using the ComBat algorithm. **(B)** Principal component analysis showing the distribution differences of different LUAD cohorts after removing batch effects using the ComBat algorithm. LUAD, lung adenocarcinoma.

Supplementary Figure 2
**(A)** Empirical cumulative distribution function diagram and **(B)** delta area diagram showing the results of consistent clustering based on the CIBERSORT results, where k represents the number of subgroups. **(C)** Consensus matrix presenting the clustering stability of hierarchical clustering for k = 3. **(D-F)** Volcano plots showing the DEGs between different TME subgroups. Red dots represent upregulated genes and green dots represent downregulated genes. **(G)** GO enrichment analysis of the 146 DEGs derived from the three TME subgroups. DEGs, differentially expressed genes. TME, tumor environment. GO, gene ontology.

Supplementary Figure 3 Principal component analysis showing the distribution differences between the LATPS-high and LATPS-low subgroups of the **(A)** training, **(B)** test, and **(C)** total cohorts. The distribution of **(D)** StromalScore, **(E)** ImmuneScore, and **(F)** ESTIMATEScore between the LATPS-high and LATPS-low subgroups. Statistical significance was assessed using the Mann-Whitney U test. The distribution of TIDE of patients with LUAD patients in the **(G)** training, **(H)** test, and **(I)** total cohorts. LATPS, LUAD tumor microenvironment prognostic signature; TIDE, tumor immune dysfunction and exclusion; LUAD, lung adenocarcinoma.

Supplementary Figure 4
**(A)** Distribution of the LATPS score in patients with different response status to anti-PD-1 therapy of NSCLC in GSE126044. **(B)** ROC analysis of the LATPS to predict an anti-PD-1 response. NSCLC, non-small cell lung cancer; PD-1, programmed cell death 1; ROC, receiver operating characteristic.

Supplementary Figure 5 Calibration curve for predicting overall survival at 1, 3, and 5 years in **(A)** test and **(B)** total cohorts. **(C-H)** Time-dependent ROC curves analysis of the nomogram and clinicopathological factors to predict 1-, 3-, and 5-year overall survival in **(C-E)** test and **(F-H)** total cohorts. ROC, receiver operating characteristic.
