# Supplementary figures and images for: LATPS, a novel prognostic signature based on tumor microenvironment of lung adenocarcinoma to better predict survival and immunotherapy response
Source: Front Immunol. 2022 Nov 24;13:1064874. doi: 10.3389/fimmu.2022.1064874 (PMC9729252; doi:10.3389/fimmu.2022.1064874)

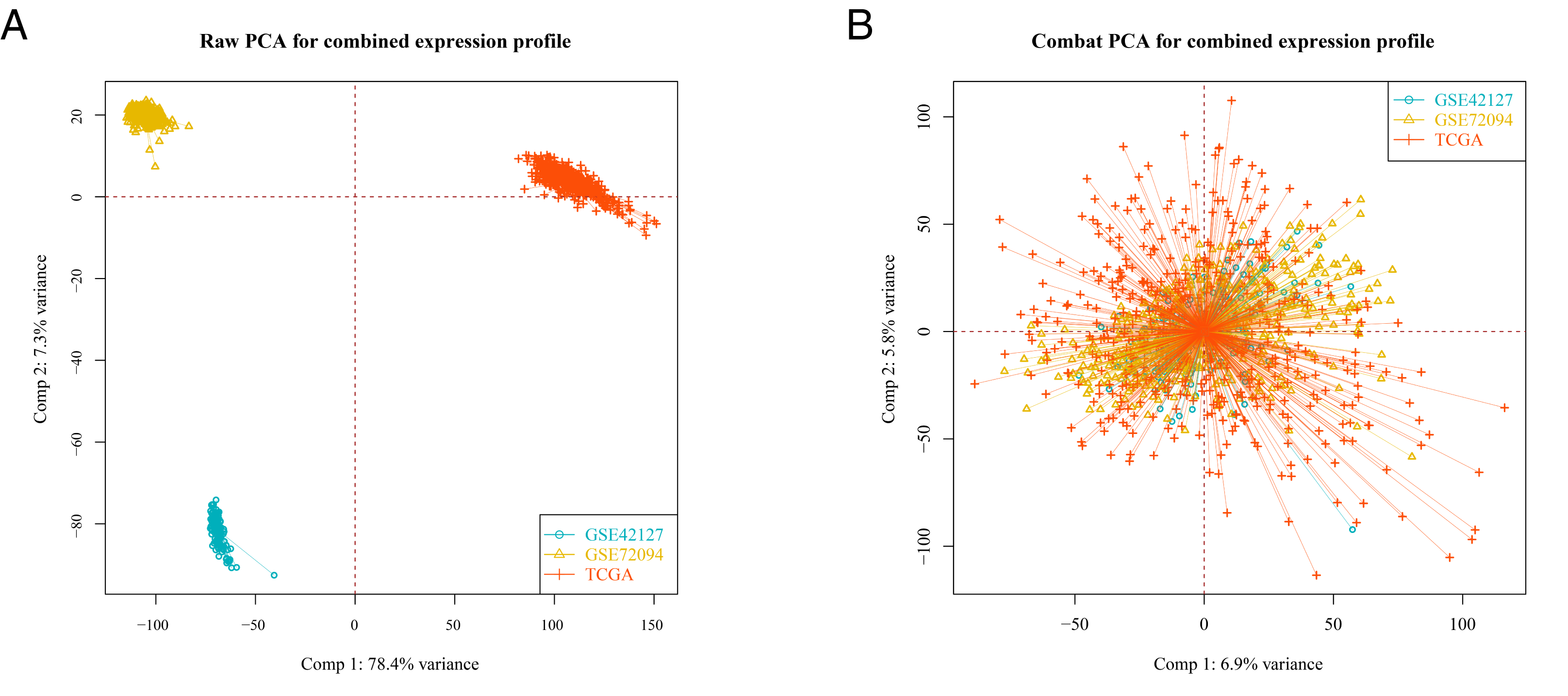

Supplement: Supplementary Figure 1 — (A) Principal component analysis showing the distribution differences of different LUAD cohorts before removing batch effects using the ComBat algorithm. (B) Principal component analysis showing the distribution differences of different LUAD cohorts after removing batch effects using the ComBat algorithm. LUAD, lung adenocarcinoma. [file Image_1.tif]

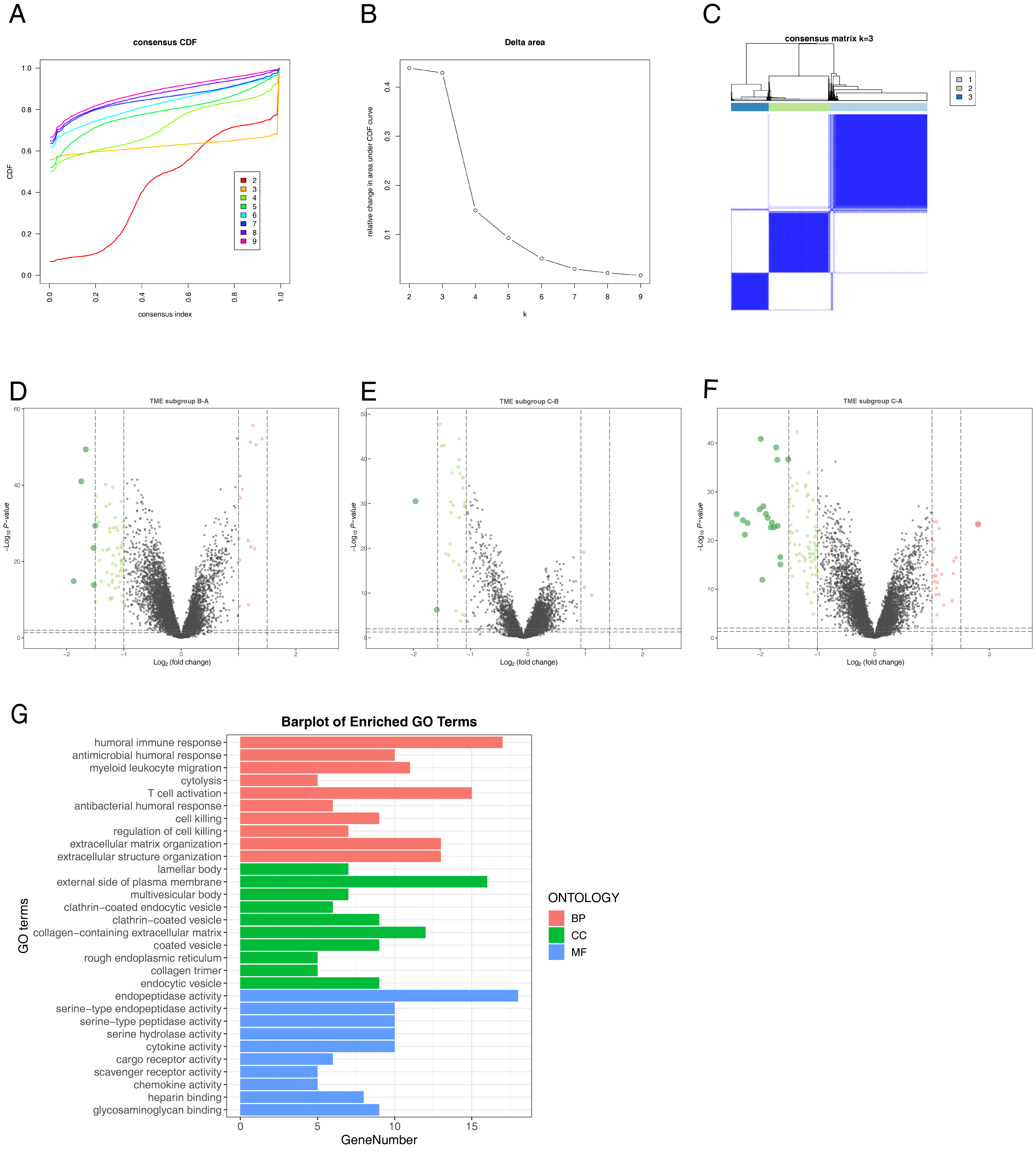

Supplement: Supplementary Figure 2 — (A) Empirical cumulative distribution function diagram and (B) delta area diagram showing the results of consistent clustering based on the CIBERSORT results, where k represents the number of subgroups. (C) Consensus matrix presenting the clustering stability of hierarchical clustering for k = 3. (D-F) Volcano plots showing the DEGs between different TME subgroups. Red dots represent upregulated genes and green dots represent downregulated genes. (G) GO enrichment analysis of the 146 DEGs derived from the three TME subgroups. DEGs, differentially expressed genes. TME, tumor environment. GO, gene ontology. [file Image_2.tif]

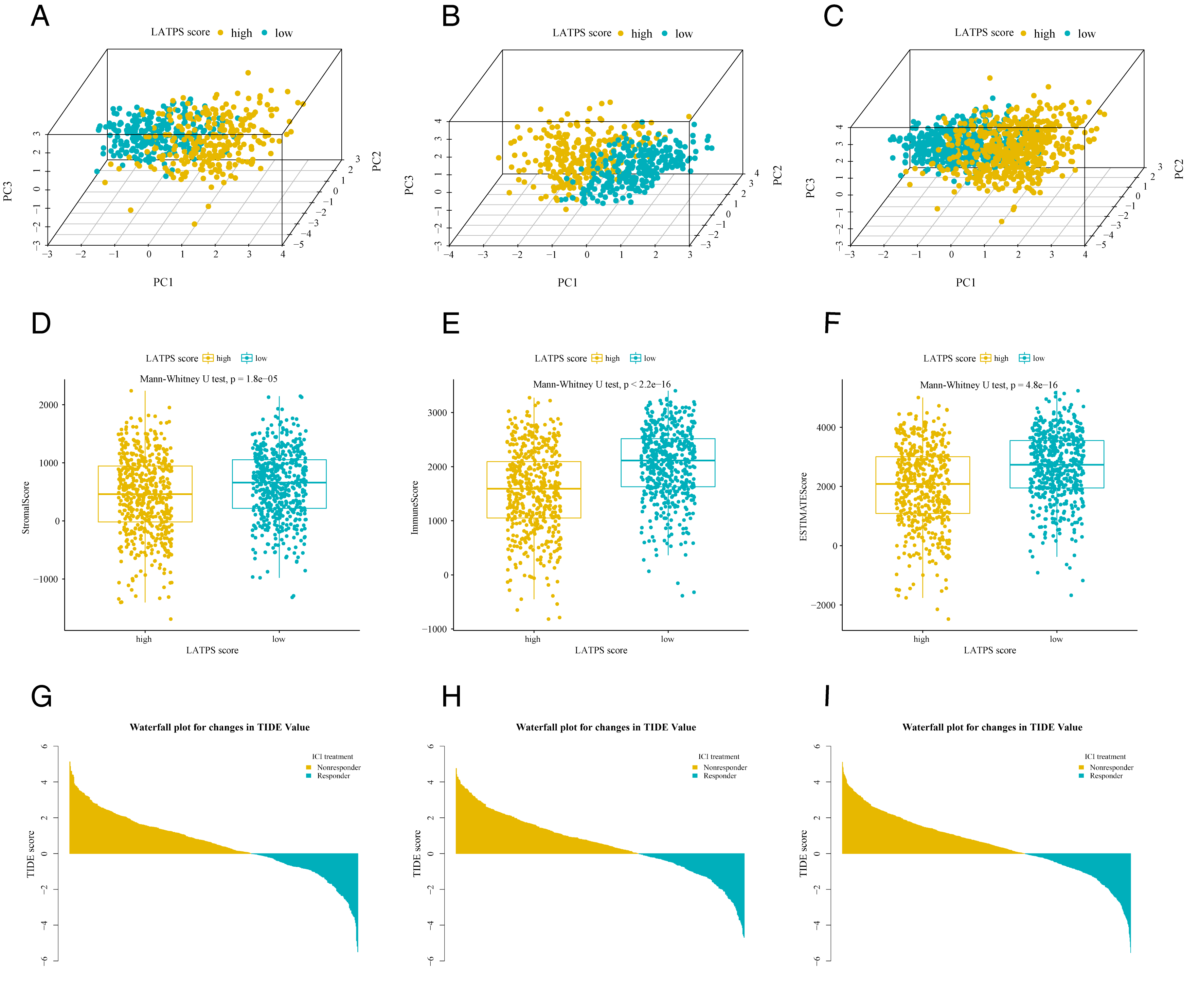

Supplement: Supplementary Figure 3 — Principal component analysis showing the distribution differences between the LATPS-high and LATPS-low subgroups of the (A) training, (B) test, and (C) total cohorts. The distribution of (D) StromalScore, (E) ImmuneScore, and (F) ESTIMATEScore between the LATPS-high and LATPS-low subgroups. Statistical significance was assessed using the Mann-Whitney U test. The distribution of TIDE of patients with LUAD patients in the (G) training, (H) test, and (I) total cohorts. LATPS, LUAD tumor microenvironment prognostic signature; TIDE, tumor immune dysfunction and exclusion; LUAD, lung adenocarcinoma. [file Image_3.tif]

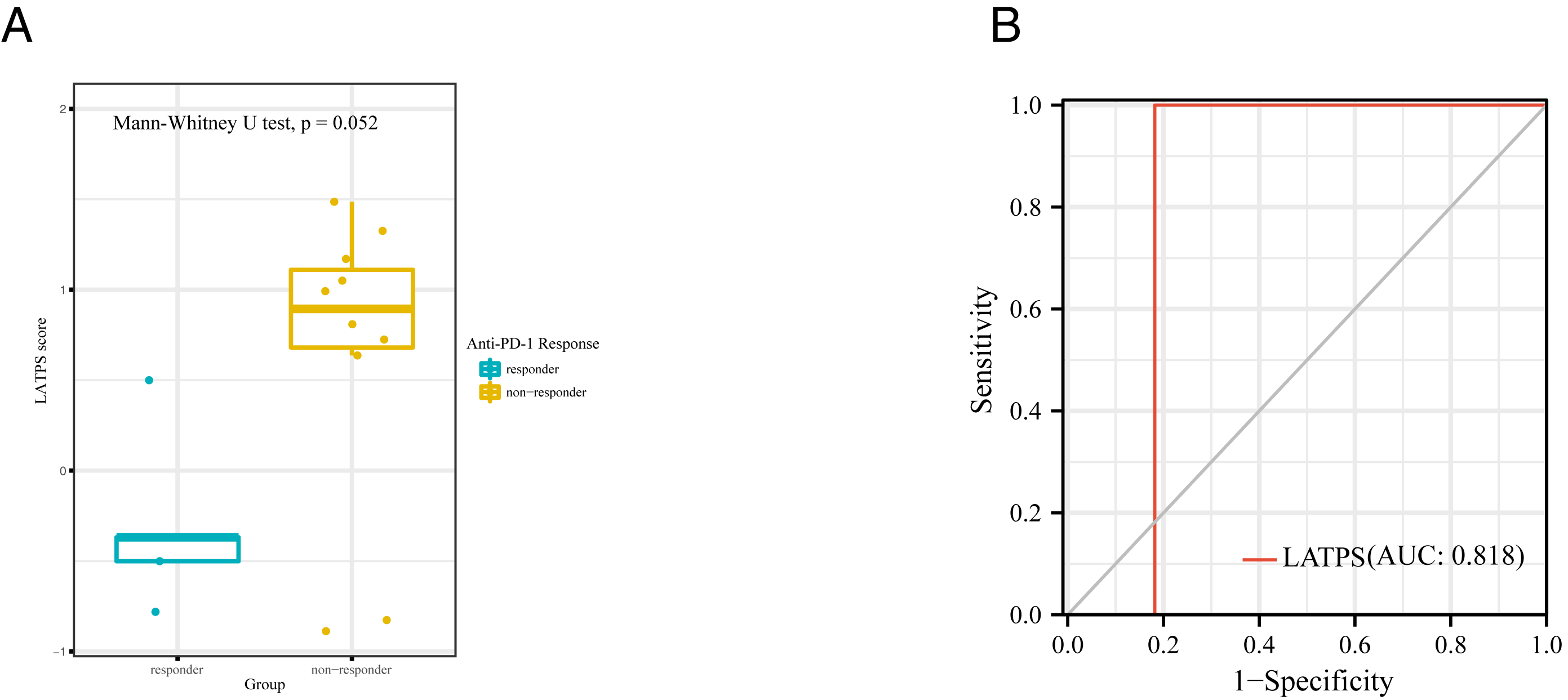

Supplement: Supplementary Figure 4 — (A) Distribution of the LATPS score in patients with different response status to anti-PD-1 therapy of NSCLC in GSE126044. (B) ROC analysis of the LATPS to predict an anti-PD-1 response. NSCLC, non-small cell lung cancer; PD-1, programmed cell death 1; ROC, receiver operating characteristic. [file Image_4.tif]

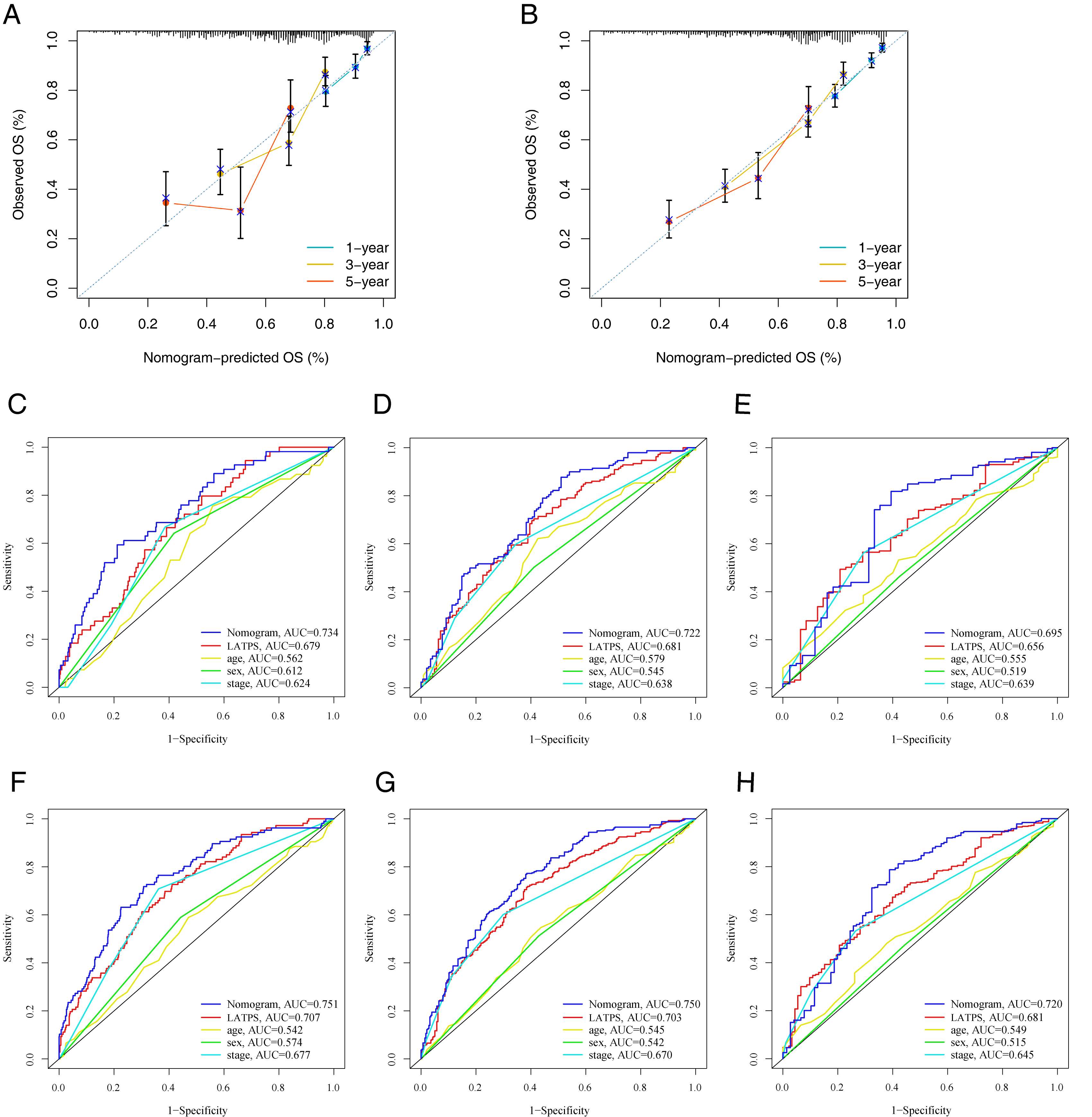

Supplement: Supplementary Figure 5 — Calibration curve for predicting overall survival at 1, 3, and 5 years in (A) test and (B) total cohorts. (C-H) Time-dependent ROC curves analysis of the nomogram and clinicopathological factors to predict 1-, 3-, and 5-year overall survival in (C-E) test and (F-H) total cohorts. ROC, receiver operating characteristic. [file Image_5.tif]
